# Differential HER2 Expression Across Feline Nasal Carcinoma and Its Relationship with Proliferation and p53 Status

**DOI:** 10.3390/vetsci13030212

**Published:** 2026-02-25

**Authors:** Maral Anjomanibenisi, Ginevra Martinoli, Michele Olei, Barbara Bacci, Barbara Brunetti

**Affiliations:** Department of Veterinary Medical Sciences, University of Bologna, 40064 Bologna, Italy; maral.anjomanibenis2@unibo.it (M.A.); ginevra.martinoli2@unibo.it (G.M.); michele.olei@studio.unibo.it (M.O.); barbara.bacci@unibo.it (B.B.)

**Keywords:** feline nasal carcinoma, HER2, p53, Ki-67, PCNA, immunohistochemistry, digital pathology

## Abstract

Feline nasal carcinomas are rare but clinically aggressive neoplasms. This study characterizes their histopathological features and evaluates HER2, p53, Ki-67, and PCNA expression using immunohistochemistry and digital image analysis, aiming to provide a comprehensive biological characterization with potential prognostic and therapeutic implications. Tumors were classified into adenocarcinomas (AC) and non-adenocarcinomas (non-AC). Among the 23 cases examined, adenocarcinoma was the most common subtype (17 cases). HER2 was scored as 3+ in 7 cases, 2+ in 8 cases, 1+ in 5 cases, and 3 cases were scored 0. A statistically significant association was found between histological type and HER2 expression (Fisher’s exact test, *p* = 0.02), with a higher prevalence of HER2 positivity in adenocarcinomas. Evaluation of p53 expression according to histological grouping showed a trend toward significance (*p* = 0.0593), with p53 positivity observed exclusively in non-AC. The Ki-67 index had a median of 4.4 (min 0.5, max 21.06), and the PCNA index had a median of 82.26 (min 19.55, max 100). No significant associations were identified between the Ki-67 labeling index and HER2 expression, histotype, and the inflammatory infiltrate. Finally, Pearson correlation analysis revealed no significant correlation between Ki-67 and PCNA indices (*p* = 0.32). The overexpression of HER2 lays the groundwork for the possible use of anti-HER2 targeted drugs in this tumor type, particularly in adenocarcinomas. These findings provide baseline immunohistochemical data for feline nasal carcinomas and highlight HER2 as a relevant biomarker for future diagnostic and therapeutic research.

## Simple Summary

Feline nasal carcinomas are rare, locally aggressive malignant tumors that typically occur in older cats. Information on their biological and immunohistochemical features is still limited. In this study, 23 feline nasal carcinomas were evaluated to describe their histological characteristics and the expression of selected immunohistochemical markers, including Ki-67, PCNA, p53, and HER2. Most tumors were adenocarcinomas, particularly of the acinar subtype. Ki-67 expression was generally low, while PCNA showed high positivity in all cases. p53 expression was uncommon. Notably, HER2 overexpression was detected in approximately one-third of the tumors, particularly in adenocarcinomas. These results provide baseline immunohistochemical data for feline nasal carcinomas and identify HER2 as a potential biomarker for future diagnostic and therapeutic investigations.

## 1. Introduction

Nasal cavity and paranasal sinus tumors are rare in cats but clinically significant, typically affecting older animals and often exhibiting aggressive local behavior. In contrast, distant metastasis is considered uncommon [1]. Sinonasal neoplasms account for 1–8.4% of all feline tumors, and approximately 90% are malignant [2], with lymphoma (51.1%) and adenocarcinoma (38.4%) being the most frequent diagnoses, followed by squamous cell carcinomas. At the same time, sarcomas represent the least common neoplasm [3]. Clinical signs often resemble those observed in dogs and include dyspnea, nasal discharge, sneezing, and epistaxis. Cats with nasal tumours frequently undergo euthanasia, reflecting the poor prognosis associated with neoplastic disease [4]. Despite their clinical relevance, the clinicopathological characterization and classification of feline nasal tumors remain limited. The absence of well-established prognostic biomarkers for feline nasal carcinomas represents a significant clinical challenge, limiting the ability to accurately predict tumor behavior and clinical outcome. Most published studies rely on classification schemes adapted from canine pathology [5], potentially introducing inconsistencies related to species differences and small sample sizes. Accordingly, definitive diagnosis currently relies on histopathological and immunohistochemical evaluation of nasal biopsies, as routine clinical and imaging findings cannot reliably distinguish neoplastic from non-neoplastic conditions [1]. Radiotherapy, administered using either hypofractionated or conventionally fractionated protocols, represents the main therapeutic option for feline nasal carcinomas. Hypofractionated protocols provide practical benefits but carry an increased risk of late adverse effects [6,7]. Reported survival times vary considerably across studies, ranging from approximately 2 to 36 months for conventionally fractionated radiotherapy protocols, while hypofractionated treatments have been associated with median survival times of about 382 to 450 days [6,7,8].

Nevertheless, comprehensive studies evaluating prognostic factors and treatment outcomes in feline nasal carcinoma are still scarce. The application of immunohistochemistry (IHC) in feline nasal mucosal carcinomas has been reported only sporadically, evaluating COX-2, cytokeratin, and S-100 [1,2,9]. Ki-67 is a non-histone nuclear protein present in actively dividing cells throughout all phases of the cell cycle, except for the quiescent G0 phase [10]. Ki-67 has been evaluated in feline nasal carcinomas, primarily for prognostic assessment following radiotherapy, and it was found that cats with high Ki-67 expression had significantly longer survival times than those with lower Ki-67 levels [11]. One drawback of this antigen is that prolonged fixation of the sample in formalin can significantly alter the expression of this marker negatively [12,13]. PCNA, or proliferating cell nuclear antigen, is another nuclear non-histone protein essential for DNA replication. In veterinary and human oncology, it has been little used in recent years because it is further involved in DNA excision repair as well as in cell cycle control, chromatin assembly, and transcriptional processes [14]. Data on PCNA expression in feline nasal carcinomas are not present. In canine nasal adenocarcinoma, overexpression and nuclear accumulation of p53 protein have been reported, suggesting a potential role of p53 dysregulation in tumor pathogenesis [15]. However, this has not been directly substantiated by original peer-reviewed studies. P53, a key tumor suppressor protein, is essential for cell cycle control and the maintenance of genomic integrity. and has been implicated in the progression of epithelial tumors across different species. Alterations of the p53 pathway have been reported in feline oncology [16,17,18]; however, to the best of our knowledge, p53 expression has not been investigated explicitly in feline nasal mucosal carcinomas. Similarly, HER2 is a transmembrane receptor involved in epithelial cell growth and differentiation. It represents an established prognostic and therapeutic target in human oncology and has also been identified as a biologically and prognostically relevant target in canine epithelial tumors [19,20,21,22]. In cats, HER2 expression has been primarily investigated in mammary carcinomas and, more recently, in pulmonary carcinomas [23,24]. The evaluation of p53 and HER2 expression in feline nasal carcinomas may therefore provide insight into shared oncogenic pathways, strengthen comparative oncology approaches, and identify potential prognostic or therapeutic implications.

This study aimed to evaluate the immunohistochemical expression of Ki-67, PCNA, p53, and HER2 in feline nasal mucosal carcinomas and to generate baseline histopathological and immunohistochemical data for this tumor type, with the purpose of providing a comprehensive biological characterization to support future diagnostic, prognostic, and therapeutic investigations. Ki-67 and PCNA were assessed using QuPath digital image analysis to improve accuracy, standardization, and reproducibility. The results of this study may contribute to a better biological characterization of feline nasal carcinomas and support future diagnostic, prognostic, and therapeutic investigations.

## 2. Materials and Methods

### 2.1. Case Selection

Twenty-three cases of feline nasal carcinomas were collected from the Pathology Service of the Department of Veterinary Medical Sciences of the University of Bologna and from the “I Portoni Rossi” Veterinary Hospital in Zola Predosa, which has its own histology laboratory. All cases were derived from biopsies. The cases were selected over 10 years (2014–2024), excluding those in which the tissue section measured less than 5 mm in total diameter. The initial dataset consisted of histological sections of formalin-fixed, paraffin-embedded (FFPE) material, subsequently stained with Hematoxylin and Eosin (H&E). Only carcinomas arising from the nasal mucosa were included, while carcinomas of the nasal planum were excluded. Clinical information for these cases was retrieved from submission forms or medical records.

### 2.2. Histopathological Evaluation

All 23 H&E-stained cases were reviewed by conventional microscopy to verify the diagnosis, and a morphological description was provided. The cases were classified according to the histological system proposed by Mukaratirwa et al. [1], integrated with Wilson’s [25] classification of canine sinonasal carcinoma. The carcinomas were classified into adenocarcinomas (acinar, papillary, tubulopapillary, and cystic subtypes), squamous cell carcinoma, transitional cell carcinoma, and undifferentiated carcinoma.

### 2.3. Immunohistochemistry

FFPE tissue sections were incubated at 60 °C for one hour and deparaffinized in X-free (2 × 15 min), followed by a 2 min rinse in 100% ethanol. Endogenous peroxidase activity was blocked using 3% H_2_O_2_ in methanol for 30 min. Sections were rehydrated through graded ethanol (100%, 90%, and 70%; 2 min each) and rinsed in distilled water. Antigen retrieval was performed in a microwave oven (750 W) using citrate buffer (pH 6), followed by cooling for 20 min and washing in Tris-buffered saline (TBS). Non-specific binding was blocked with 10% normal goat serum in phosphate-buffered saline (PBS) for 30 min. Primary antibodies were applied at validated dilutions and incubated overnight at 4 °C or for 90 min at 37 °C. Details of the antibodies used for immunohistochemical analysis, including clone, manufacturer, dilution, and antigen retrieval, are reported in Table 1. For each antibody, appropriate positive control tissues with known target expression were included and processed in parallel with the study samples. Specifically, for HER2, a case of feline mammary carcinoma with confirmed HER2 overexpression (3+) was used as a positive control. For p53, a feline squamous cell carcinoma with documented nuclear immunoreactivity for p53 served as the positive control. For Ki-67 and PCNA, feline intestinal mucosa served as the positive control. Negative controls were obtained by omitting the primary antibody. Sections were then washed and incubated with the corresponding secondary antibody for 30 min. After TBS washes, the avidin–biotin complex (ABC) reagent (Vector Laboratories, Burlingame, CA, USA) was applied for 30 min. Chromogenic detection was performed with 3,3′-diaminobenzidine (DAB; Histoline Laboratories, Milan, Italy) or 3-amino-9-ethylcarbazole (AEC; Vector Laboratories, Burlingame, CA, USA), and color development was monitored under a microscope. Sections were rinsed and counterstained with Harris hematoxylin (DAB) or Mayer’s hematoxylin (AEC). DAB-stained sections were dehydrated through graded ethanol and cleared in X-free, whereas AEC-stained sections were kept ethanol-free to preserve chromogen integrity. Slides were mounted using mounting medium (Bio-Optica, Milan, Italy) (DAB) or aqueous mounting gel (Bio-Optica, Milan, Italy) (AEC) and dried in the dark.

### 2.4. Immunohistochemical Evaluation System

#### 2.4.1. HER2

In the absence of validated feline-specific scoring systems, HER2 expression was evaluated immunohistochemically by assessing positivity in neoplastic cells and following the ASCO/CAP guidelines [26], using a 10% threshold as the reference criterion (complete, intensive membranous staining in more than 10% of neoplastic cells). Immunoreactivity to HER2 was divided into the following classes: 3+ (positive), 2+ (equivocal), and 0 and 1+ (negative). HER2 immunoreactivity was also evaluated according to the consensus on HER2 alterations in Non-Small Cell lung cancer in humans [27], already utilized in veterinary oncology [28], with 0 and 1+ considered negative and 2+ and 3+ considered HER2-positive.

#### 2.4.2. P53

Immunohistochemical assessment of p53 expression was conducted by evaluating nuclear staining exclusively in neoplastic cells. In this study, tumors were classified as p53-positive when the proportion of neoplastic cells expressing mutated p53 exceeded 10% [29].

#### 2.4.3. Ki-67

Immunohistochemical evaluation of Ki-67 expression was performed by assessing nuclear positivity in neoplastic cells only. In contrast to p53, Ki-67 slides underwent both conventional microscopic assessment and digital scanning using the Grundium Ocus® scanner (Grundium, Tampere, Finland) (20×, 0.25 µm/pixel). Whole-slide images were saved in “.svs” format and labeled with the case identifier. Digital images were subsequently imported into QuPath software v0.6.0 (University of Edinburgh, Edinburgh, UK) for automated calculation of the Ki-67 index.

#### 2.4.4. PCNA

Immunohistochemistry for Proliferating Cell Nuclear Antigen (PCNA) was also performed on cases to evaluate PCNA expression in neoplastic cells. Only nuclear staining was considered specific and recorded as positive. Slides stained for PCNA underwent both conventional microscopic evaluation and digital scanning using the Grundium Ocus® scanner (Grundium, Tampere, Finland) (20×, 0.25 µm/pixel). Whole-slide images were saved in “.svs” format and labeled with the corresponding case identifier. Digital images were subsequently imported into QuPath software v0.6.0 (University of Edinburgh, Edinburgh, UK) for automated calculation of the PCNA index.

### 2.5. Image Analysis Using the QuPath Software

Ki-67 and PCNA slides were analyzed using QuPath software v0.6.0 (University of Edinburgh, Edinburgh, UK). Separate projects were created for AEC- and DAB-stained slides to facilitate accurate color recognition. For each slide, a 1 mm^2^ neoplastic area exhibiting the highest staining intensity and including at least 500 tumor cells was manually selected, excluding stroma, inflammatory cells, necrotic areas, and artifacts, as described by Bacci et al. [30]. This targeted hotspot approach was chosen to capture the tumor’s peak proliferative activity and to avoid dilution of the proliferative index that can occur with whole-slide averaging. Furthermore, this method was necessitated by the nature of feline nasal biopsies, which are frequently small and subject to tissue exhaustion during sequential immunohistochemical sections. To ensure a reliable distinction between neoplastic and reactive cells, the software was trained to discriminate between positive and negative neoplastic nuclei based on specific histomorphological criteria (e.g., nuclear atypia and pleomorphism). For each slide, training was performed on multiple sample annotations using the ‘Positive cell detection’ tool by adjusting optical density sum, nuclear, and intensity parameters. To compensate for any limitations in the initial training label set, a multi-step validation was implemented: once the sample annotation was satisfactory, the analysis was applied, and the results were subsequently inspected by two authors. Parameters were further refined, or manual corrections were applied to minimize misclassification, ensuring that reactive cells and artifacts were excluded and only neoplastic nuclei were included in the final quantification.

All proliferation indices were exported to Microsoft Excel (Microsoft Corporation, Redmond, WA, USA) for statistical analysis. Although digital quantification of p53 expression could be performed, it offers limited advantages in this context. The extensive time required for whole-slide scanning and subsequent computational analysis via QuPath outweighs the benefits when compared to the established efficiency and reliability of semiquantitative visual assessment. Regarding HER2 quantification, digital image analysis—including the use of QuPath integrated with Cellpose software (Broad Institute of MIT and Harvard, Cambridge, MA, USA) was initially attempted; however, it failed to yield optimal results due to significant challenges in membrane segmentation and signal-to-noise optimization. Since alternative specialized software platforms were not available in our facility to overcome these technical limitations, we opted for traditional semiquantitative microscopic assessment.

### 2.6. Statistical Analysis

All statistical analyses were performed with R software, version 4.5.2 (R Foundation for Statistical Computing, Vienna, Austria). Categorical data were summarized as frequencies and percentages, and continuous data as mean, median, standard deviation (SD), and range. Correlations between continuous variables were assessed using Pearson’s test, while Fisher’s exact test was employed to analyze associations between categorical variables. The relationship between continuous and categorical variables was examined using one-way ANOVA or, when necessary, the Kruskal–Wallis test. A *p*-value < 0.05 was considered statistically significant.

## 3. Results

### 3.1. Patient and Tumor Characteristics

A total of 23 cats were included in the study: 6 neutered males, 5 intact males, 7 spayed females, and 5 intact females. The mean age was 13.78 ± 3.10 years (range: 8–19 years). Most subjects were European Shorthair cats (20/23, 86.95%), while one Ragdoll, one Birman, and one Siamese were also represented (4.35% each). Clinical signs reported at presentation included a nasal mass, nasal necrosis and hemorrhage, sneezing, nasal discharge, rhinitis, facial deformity, dyspnea, and mucous material. Most cats showed more than one clinical sign. Tumor extension into the nasopharynx was observed in 9 of 23 cases (39.13%). Neoplastic masses were located in the right nasal cavity in 8 cases (34.78%), in the left cavity in 4 cases (17.39%), and bilaterally in 5 cases (21.74%); the location was not specified in 6 cases (26.09%) (Supplementary Table S1). Two cases had suspected pulmonary metastases reported in the submission forms; however, these were excluded from statistical analysis due to the lack of a confirmatory histological diagnosis.

### 3.2. Histological Diagnosis

**Figure 1:**
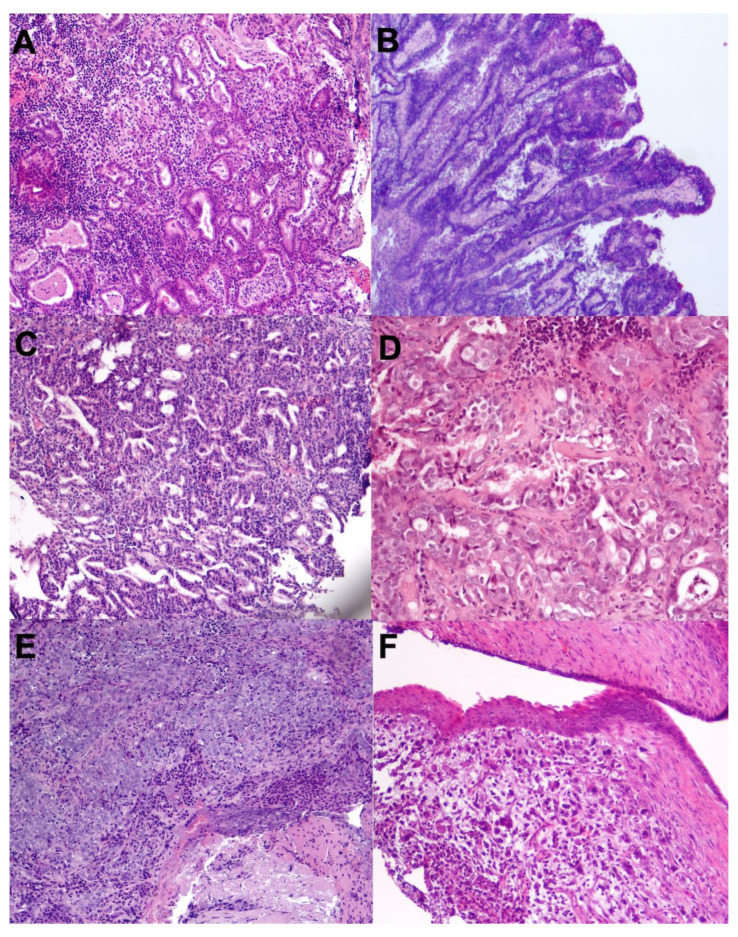
Histological features of the different types of feline nasal carcinomas (H&E). (**A**) Acinar adenocarcinoma (10×); (**B**) Papillary adenocarcinoma (4×); (**C**) Tubulopapillary adenocarcinoma (10×); (**D**) Transitional carcinoma (20×); (**E**) Squamous cell carcinoma (10×); (**F**) Undifferentiated carcinoma (10×).

The most frequently diagnosed carcinoma was adenocarcinoma (17 cases). The most common AC subtype was acinar adenocarcinoma (13 cases), followed by papillary adenocarcinoma and tubulopapillary adenocarcinoma, each with 2 cases. Six non-AC were detected, including 2 transitional carcinomas, 2 squamous cell carcinomas, and 2 undifferentiated carcinomas (Figure 1).

A lymphoplasmacytic inflammatory infiltrate was observed in all tumors, most commonly in moderate (12 out of 23) and mild (7 out of 23) quantities.

### 3.3. Immunohistochemical and Image Analysis

**Figure 2:**
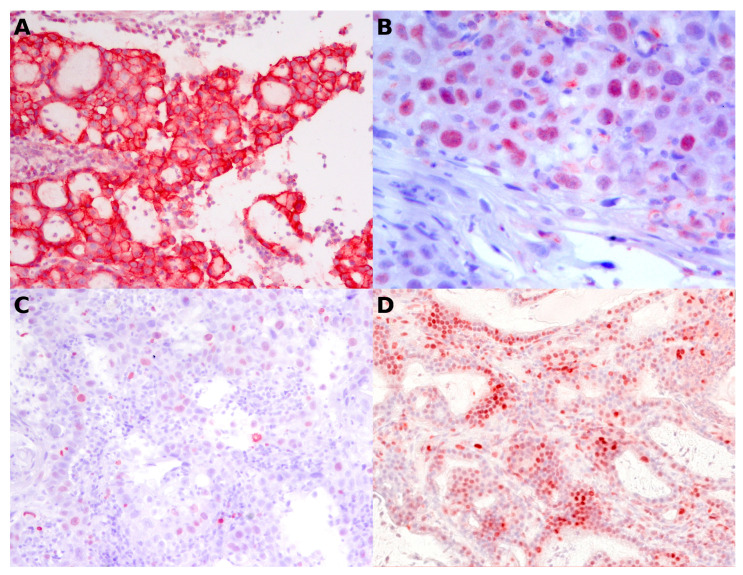
Immunohistochemical staining patterns of HER2 (**A**), p53 (**B**), Ki-67 (**C**), and PCNA (**D**) in feline nasal carcinoma. (**A**) HER2 showing strong membranous immunoreactivity in neoplastic epithelial cells (20×). (**B**) p53 exhibiting nuclear immunopositivity in a subset of tumor cells (40×). (**C**) Ki-67 highlighting proliferating neoplastic cells with variable nuclear labeling (20×). (**D**) PCNA nuclear immunoreactivity in neoplastic cells (20×).

**Figure 3:**
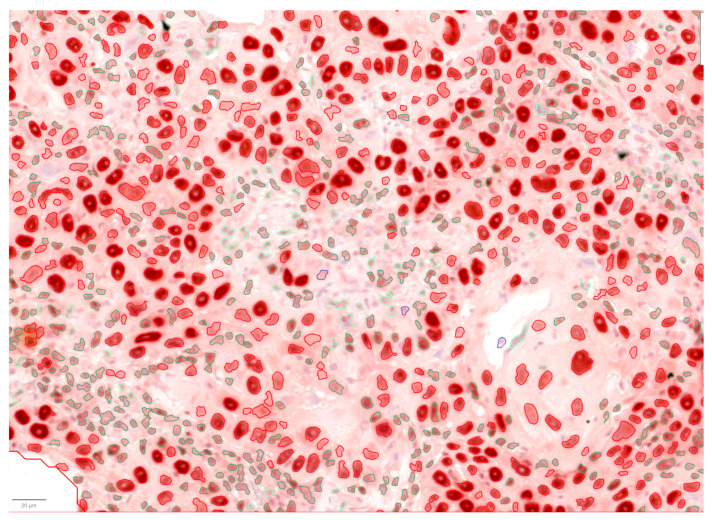
Representative digital image analysis for PCNA index and positivity detection, including automated nuclear detection and classification of tumor nuclei. Immunopositive tumor nuclei outlined in red, immunonegative tumor nuclei outlined in blue, and stromal nuclei outlined in green.

Immunohistochemical analysis demonstrated expression of the nuclear markers p53, PCNA, and Ki-67, as well as the membrane marker HER2. HER2 staining resulted in 3 cases scoring 0, 5 scoring 1+, 8 scoring 2+, and 7 scoring 3+. Scores 0, 1+, and 2+ (16 cases) were classified as negative, whereas score 3+ (7 cases) was considered positive following the ASCO/CAP guidelines [26], instead scores 0 and 1+ (8 cases) were classified as negative, and 2+ and 3+ (15 cases) as positive following the other method [27,28]. Using the anti-p53 antibody, 21 cases were negative, and two were positive (8.7%). One case showing exclusively cytoplasmic staining was included among the negatives, as the staining was considered non-specific. Ki-67 (clone MIB-1) expression showed variable proliferative activity, with a median of 4.4% (range: 0.5–21.06%). Eight cases were non-assessable despite repeated testing, with the positive control testing positive; these cases were considered false negatives. Among evaluable samples, proliferation was classified as low or high based on 4% as the threshold (median of 4.4%). The PCNA index had a mean of 75.17 and a median of 82.26 (min 19.55, max 100). All carcinomas were PCNA-positive and showed much higher PCNA labeling indices than Ki-67 labeling indices. Representative immunohistochemical staining patterns for the used antibodies are shown in Figure 2. A representative example of digital image analysis used to assess the PCNA labeling index is shown in Figure 3 and summarized in Table 2.

### 3.4. Statistical Results

**Figure 4:**
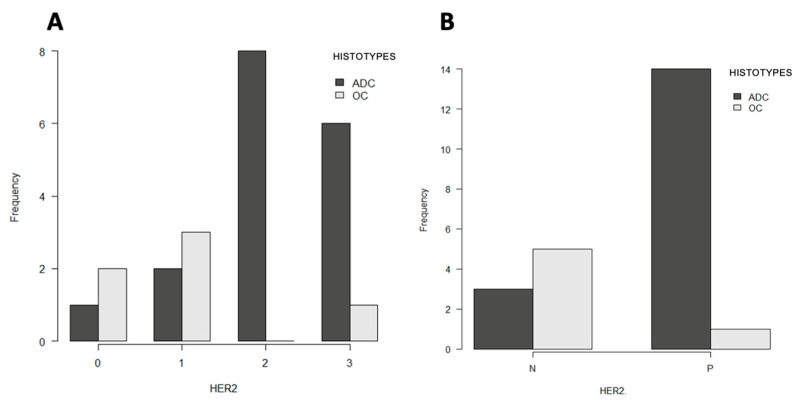
Association between histological type and HER2 immunohistochemical expression in feline nasal carcinomas. (**A**) Distribution of HER2 scores (0, 1+, 2+, 3+) in AC and non-AC. AC cases show a higher frequency of 2+ and 3+ HER2 scores, whereas non-AC cases are mainly distributed in the lower score categories (0, 1+). (**B**) Comparison of HER2 status classified as negative (N) (0/1+) or positive (P) (2+/3+) between AC and non-AC. HER2 positivity is predominantly observed in AC, whereas non-AC cases are mostly HER2-negative.

Statistical analysis was performed to investigate potential associations among HER2, p53, Ki-67, and PCNA indices and clinical-pathological data. Regarding the histotype, AC and non-AC were used as categories. No statistically significant differences in the Ki-67 index were observed between the two histotypes (AC and non-AC) (Kruskal–Wallis test, *p* = 0.80). Similarly, the PCNA index did not differ significantly between the two groups (*p* = 0.16). There was a statistically significant difference in histological type and HER2 expression (Fisher’s exact test, *p* = 0.02) (Figure 4A). Considering only the 3+ HER2 score as positive, no statistically significant difference was observed between AC and non-AC (Fisher exact test *p* = 0.62). When considering both 2+ and 3+ score as HER2-positive cases, there was a statistically significant difference between the two histological groups (Fisher exact test *p* = 0.0086) (Figure 4B). Regarding specific histotype and HER2 expression, acinar adenocarcinomas showed heterogeneous HER2 scores (3+, 2+, and 1+), whereas undifferentiated carcinomas were uniformly HER2 1+, and squamous cell carcinomas were HER2-negative (score 0).

Expression of p53 was compared between AC and non-AC, and a value just short of significance was obtained (Fisher’s exact test, *p* = 0.0593), as both p53-positive cases were in the non-AC category (1 undifferentiated and 1 squamous cell carcinoma).

The relationship between the Ki-67 labeling index and HER2 immunohistochemical score was evaluated using the Kruskal–Wallis test, with no statistically significant differences observed among HER2 (3+ as positive and 0, 1+, 2+ as negative) (*p* = 0.637), or HER2 (2+ and 3+ as positive, and 0 and 1+ as negative) (*p* = 0.40) or HER2 (group as 0, 1+, 2+, or 3+) (*p* = 0.32). Similarly, the Ki-67 labeling index and the inflammatory infiltrate showed a *p*-value of 1. The relationship between the PCNA labeling index and HER2 score was evaluated using the Kruskal–Wallis test, with no statistically significant differences observed among HER2 scores when 3+ was considered positive and 0, 1+, and 2+ were considered negative (*p* = 0.16). A similar result was found for HER2, with 2+ and 3+ considered positive and 0 and 1+ negative (*p* = 0.09), while a statistically significant difference was found between PCNA labelling index and the HER2 group (0, 1+, 2+, 3+). (*p* = 0.02). A Pearson correlation was tested between the Ki-67 and PCNA indices, yielding *p* = 0.32 and no significant correlation.

## 4. Discussion

Feline nasal carcinomas are uncommon but clinically significant malignant neoplasms, including adenocarcinomas, squamous cell carcinomas, and undifferentiated carcinomas [31]. These tumors are typically associated with early clinical signs such as nasal discharge and/or epistaxis, which, as the disease progresses, may be accompanied by dyspnea, sneezing, and facial profile deformity [32]. Usually, 2 to 4 weeks elapse between the onset of clinical signs and the diagnosis of nasal carcinoma, although in some cases several years may pass. The most affected cats are at least 6 years old, with the frequency increasing in individuals approaching 12 years of age [33]; in the present study, the mean age was approximately 14 years. Diagnosis begins with macroscopic examination and is completed through surgical collection of the neoplastic tissue, followed by histological and/or immunohistochemical analysis, sometimes supported by radiographic imaging [34]. Treating a nasal carcinoma is challenging, mainly when the tumor displays aggressive and infiltrative behavior. Therapeutic options include surgery—typically insufficient as a sole treatment—chemotherapy, and more commonly, radiotherapy, which has demonstrated the most favorable prognostic outcomes [4,34,35,36]. In the present study, 23 feline nasal carcinomas were assessed and classified into six histological subtypes. The observed distribution confirms the predominance of adenocarcinomas—particularly the acinar variant—whereas all the other subtypes were less represented, reflecting their rarity in the feline species. Immunohistochemistry is particularly valuable in the diagnostic process. Among the most widely used markers in both human and veterinary oncology are Ki-67 [37,38], HER2 [24,39], and p53 [29,40,41]. In veterinary medicine, the literature concerning these markers remains limited, particularly for feline nasal carcinomas. Ki-67 has been applied to evaluate treatment response and prognosis in feline nasal adenocarcinomas treated with radiotherapy [11]. Cats with high Ki-67 expression who underwent radiotherapy had significantly longer survival times than those with low Ki-67 expression; a threshold of 40% (the median was 41.42%) was used as the cutoff for separating low and high Ki-67 expression [11]. Immunohistochemical evaluation of Ki-67 was supported by a digital, automated image analysis approach, enabling an objective and reproducible assessment of tumor proliferative activity. Compared with traditional manual evaluation, digital analysis enables quantitative assessment of all neoplastic cells within a predefined hotspot, reducing observer-dependent variability and minimizing the influence of field selection and subjective cell counting, which are known limitations of manual Ki-67 scoring. The median of our cases was 4.4% with a minimum of 0.5% and a maximum of 21.06% in acinar adenocarcinomas. The mean Ki-67 labeling index observed in the present study (4.4%) is substantially lower than the approximately 40% reported in feline nasal carcinomas [11]. This discrepancy may reflect differences in Ki-67 evaluation methodology. In the latter study, Ki-67 was assessed by manual counting of 1000 tumor cells from five randomly selected high-power fields (60×). In contrast, the present study employed a digital image analysis approach applied to a standardized tumor area of 1 mm^2^ selected within the hotspot exhibiting the highest Ki-67 immunoreactivity. Although both methods focus on highly proliferative tumor regions, digital analysis includes all neoplastic cells within the selected area, regardless of staining intensity, thereby providing a more objective and reproducible estimate of proliferative activity and potentially resulting in lower overall Ki-67 values. Therefore, direct numerical comparison of Ki-67 indices across studies using different assessment strategies should be interpreted with caution. Unfortunately, the Ki-67 index was not correlated with any of the parameters examined, and 8 of 23 cases were non-reactive, probably due to the long time the samples had spent in formalin. This loss of Ki-67 immunoreactivity represents a limitation of the study and reduces the statistical power of the proliferation analysis. PCNA was evaluated as an exploratory marker in samples non-reactive for Ki-67; however, the lack of correlation between Ki-67 and PCNA expression indicates that PCNA cannot reliably substitute Ki-67 as a proliferation index in feline nasal carcinomas. PCNA was reactive in all samples but showed a much higher positivity index than Ki-67. Although PCNA’s main role is in DNA replication, it is additionally involved in DNA excision repair, cell cycle control, chromatin organization, and RNA transcription [14,42]. In our caseload, the median PCNA index was 82.85%, which was extremely high compared to the median Ki67 index. In addition, a poor correlation between PCNA and Ki-67 labeling indices was observed (*p* = 0.32). This poor correlation between the two proliferation markers has already been documented in human medicine, indicating that PCNA immunostaining may not represent a reliable surrogate for the Ki-67 index in patients with pulmonary adenocarcinomas and breast cancer [43,44]. Given our results with PCNA, we suggest that PCNA, particularly when assessed using the PC10 clone, may not reliably reflect proliferative activity and should be interpreted with caution. Future studies using optimized fixation protocols or additional proliferation markers may help overcome these technical limitations.

p53 immunolabeling has already been investigated in feline carcinomas [17,45,46,47,48], showing a positivity of 24% to 69% of feline oral squamous cell carcinomas [17], a positivity of 13.7% to 73% in feline mammary carcinomas [45,48], and positivity in one case of conjunctival feline squamous cell carcinoma [45]. Moreover, in some of these studies, the authors used the exact clone we used [17,45]. Using the monoclonal antibody clone PAb240 [17], a good correlation between p53 immunopositivity and the presence of *TP53* gene mutations identified by next-generation sequencing in feline oral squamous cell carcinoma was reported. Unfortunately, there are no studies of p53 expression or *TP53* mutations in feline nasal mucosal carcinomas; in our caseload, only 2 of 23 cases (8.7%) were positive for p53 (1 undifferentiated and 1 squamous cell carcinoma), with a *p*-value bordering on significance. The very low number of positive cases (*n* = 2) severely limits the statistical power of this analysis. Therefore, these results should be interpreted with caution and considered exploratory. Such low positivity, although partly due to the use of a monoclonal antibody, suggests that p53 is not significantly involved in the pathogenesis of these tumors in cats. Obviously, this is only one assessment of the presence or absence of the p53 protein, since immunoreactivity alone does not reliably reflect the mutational status of the *TP53* gene. Molecular techniques, including next-generation sequencing, will be required to detect *TP53* mutations and assess their role in tumor development.

A notable finding was the detection of HER2 expression, with a considerable proportion of cases showing an immunohistochemical score of 3+. Although HER2 has been extensively investigated in human oncology, information on its expression in feline nasal tumors is lacking, and, to the best of our knowledge, HER2 expression in feline nasal carcinomas has not previously been documented. The present findings therefore provide preliminary evidence that HER2 may be overexpressed in a subset of feline nasal carcinomas. There was a statistically significant difference in HER2 expression between AC and non-AC, with adenocarcinomas showing higher HER2 expression (score 2+ and 3+). Adenocarcinomas express higher levels of HER2 than other histotypes. These findings may indicate a different pathogenesis between adenocarcinomas and other carcinomas, with a prevalent role of HER2 in adenocarcinomas. Although HER2 expression may reflect biologically relevant tumor characteristics, the lack of follow-up data in the present study precludes any direct conclusions regarding its prognostic significance. Future studies integrating HER2 expression with survival and clinical outcome data are warranted to clarify its potential prognostic role in feline nasal carcinomas. By contrast, for non-adenocarcinomas, a potential p53–related pathogenic mechanism may be considered. Further studies, including molecular confirmation of equivocal (2+) cases, are necessary to clarify the biological relevance of HER2 in the pathogenesis of these tumors. Overall, these findings provide a rationale for exploring HER2-targeted therapeutic strategies in feline nasal carcinomas, and particularly in adenocarcinomas.

Unfortunately, no correlation was found between HER2 overexpression and the Ki-67 index in our cases. Only a significant association was found between PCNA index and the various HER2 groups, in particular HER2 2+, and a lower PCNA index. This data is difficult to interpret and probably has no significance. The principal limitation of this study is the relatively small sample size, particularly for the less-represented histological variants, which restricted statistical analyses and limited the generalizability of the findings. Future studies should aim to expand the number of cases by including samples from a broader range of diagnostic laboratories and veterinary centers, ensuring a more balanced representation of tumor subtypes. Another limitation was the presence of Ki-67–negative cases, likely due to overfixation during formalin fixation. This highlights the need for optimized tissue handling procedures or the use of alternative proliferation markers in studies involving older or poorly preserved samples.

## 5. Conclusions

In conclusion, this study provides the first evidence of HER2 expression in feline nasal carcinomas, especially in adenocarcinomas, highlighting a potential biomarker for future research and a possible therapeutic target. The low and heterogeneous p53 expression underscores the need for molecular analyses to clarify its biological significance. Expanding the case series and integrating advanced molecular approaches will be essential to validate these findings and further elucidate the mechanisms underlying nasal carcinogenesis in cats.

## Figures and Tables

**Table 1:** Primary antibodies used in the study and immunohistochemical conditions.

Antibody	Clone	Host	Manufacturer	Dilution	Antigen Retrieval	Positive Control	Localization
HER2	Polyclonal (A0485)	Rabbit	Dako, Glostrup, Denmark	1:200	HIER, citrate buffer (pH 6.0)	feline Mammary carcinoma	Membranous
Ki-67	MIB-1	Mouse	Dako, Glostrup, Denmark	1:1500	HIER, citrate buffer (pH 6.0)	feline Small intestine	Nuclear
P53	PAb240	Mouse	BD Pharmingen, San Diego, CA, USA	1:200	HIER, citrate buffer (pH 6.0)	Feline squamous cell carcinoma	Nuclear
PCNA	PC10	Mouse	Cell Signaling Technology, Danvers, MA, USA	1:2000	HIER, citrate buffer (pH 6.0)	Feline small intestine	Nuclear

HIER, heat-induced epitope retrieval.

**Table 2:** Clinical features and immunohistochemical results of feline nasal carcinomas.

Case No.	Breed	Age (Years)	Sex	Histotype	HER2 Score	p53	Ki-67 Index (%)	PCNA Index (%)
1	European	13	MC	Acinar adenocarcinoma	3+	−	21.06	99.79
2	Ragdoll	16	FS	Acinar adenocarcinoma	2+	−	NA	19.55
3	European	8	FS	Papillary adenocarcinoma	3+	−	9.95	100
4	European	19	F	Undifferentiated carcinoma	1+	− *	NA	73.40
5	European	12	F	Acinar adenocarcinoma	2+	−	4.13	38.37
6	European	13	F	Acinar adenocarcinoma	2+	−	NA	28.24
7	European	18	M	Undifferentiated carcinoma	1+	+	9.03	98.52
8	Siamese	19	F	Acinar adenocarcinoma	2+	−	NA	56.68
9	European	17	M	Acinar adenocarcinoma	2+	−	NA	89.19
10	Birman	11	M	Squamous cell carcinoma	0	−	2.20	73.46
11	European	14	M	Papillary adenocarcinoma	0	−	NA	82.26
12	European	13	M	Squamous cell carcinoma	0	+	1.80	98.36
13	European	11	F	Acinar adenocarcinoma	1+	−	4.64	96.78
14	European	15	FS	Transitional carcinoma	1+	− *	8.49	84.98
15	European	8	MC	Tubulopapillary adenocarcinoma	1+	−	2.94	99.87
16	European	15	MC	Acinar adenocarcinoma	2+	−	10.23	59.87
17	European	17	FS	Acinar adenocarcinoma	2+	−	NA	84.52
18	European	12	MC	Tubulopapillary adenocarcinoma	2+	−	4.21	46.87
19	European	13	MC	Acinar adenocarcinoma	3+	−	4.40	97.88
20	European	14	FS	Acinar adenocarcinoma	3+	−	3.79	70.33
21	European	13	FS	Acinar adenocarcinoma	3+	−	0.5	66.08
22	European	10	FS	Transitional carcinoma	3+	−	5.46	99.39
23	European	16	MC	Acinar adenocarcinoma	3+	−	NA	64.53

Abbreviations: F, intact female; M, intact male; MC, castrated male; FS, spayed female; NA, not available. +: positive, and −: negative, * p53 immunoreactivity restricted to the cytoplasm, with no nuclear staining.

## Data Availability

The original contributions presented in this study are included in the article/Supplementary Material. Further inquiries can be directed to the corresponding author.

## Supplementary Materials

The following supporting information can be downloaded at: https://www.mdpi.com/article/10.3390/vetsci13030212/s1, Table S1: Inflammatory infiltrate, clinical presentation, and local involvement of feline nasal carcinomas.

## Abbreviations

The following abbreviations are used in this manuscript

ABC	Avidin–biotin complex
AC	Adenocarcinoma
non-AC	non-Adenocarcinoma
AEC	Aminoethyl carbazole
ASCO	American Society of Clinical Oncology
CAP	College of American Pathologists
COX-2	Cyclooxygenase-2
DAB	3,3′-Diaminobenzidine
FFPE	Formalin-fixed, paraffin-embedded
FS	Spayed female
H&E	Hematoxylin and eosin
HER2	Human epidermal growth factor receptor 2
HIER	Heat-induced epitope retrieval
IHC	Immunohistochemistry
M	Intact male
MC	Castrated male
NA	Not available
NGS	Next-generation sequencing
PBS	Phosphate-buffered saline
PCNA	Proliferating cell nuclear antigen
SD	Standard deviation
TBS	Tris-buffered saline
WSI	Whole-slide image
