# Supplementary material for: Differential HER2 Expression Across Feline Nasal Carcinoma and Its Relationship with Proliferation and p53 Status
Source: Vet Sci. 2026 Feb 25;13(3):212. doi: 10.3390/vetsci13030212 (PMC13030173; doi:10.3390/vetsci13030212)
Supplement: Supplementary file 1 [file vetsci-13-00212-s001.zip › vetsci-4121301-supplementary.pdf]

Table S1. Clinical presentation and local involvement of feline nasal carcinomas.

Case No.	Inflammatory Infiltrate	Clinical Signs	Nasal Cavity Involved	Nasopharynx
1	Severe	Mass	Right	No
2	Mild	Necrosis, hemorrhage, mass	Bilateral	Yes
3	Moderate	Mass	NA	Yes
4	Moderate	Sneezing, nasal discharge, facial deformity, mass	Right	Yes
5	Severe	Bloody sneezing, mass	Right	Yes
6	Moderate	Sneezing, mucous nasal discharge, facial deformity, mass	Right	Yes
7	Mild	Necrosis, hemorrhage, mass	NA	No
8	Moderate	Catarrhal hemorrhagic material, mass	Right	Yes
9	Severe	Hemorrhage, mass	NA	Yes
10	Severe	Necrosis, hemorrhage	Bilateral	No
11	Moderate	Mass	NA	No
12	Moderate	Mass	Left	No
13	Mild	Chronic rhinitis, nasal discharge, mass	Right	No
14	Mild	Hemorrhage, mass	NA	No
15	Moderate	Nasal discharge, facial profile deformity	Left	No
16	Moderate	Friable whitish mass	Bilateral	Yes
17	Moderate	Hemorrhage, mass	Right	Yes
18	Mild	Facial profile deformity	Bilateral	No
19	Moderate	Respiratory distress	Right	No
20	Moderate	Hemorrhagic nasal discharge	Left	No
21	Mild	Hemorrhage	Bilateral	No
22	Moderate	Facial profile deformity	NA	No
23	Mild	Mass	Left	NA

NA, not available.
